# On the Necessity of Accoucheurs

**Published:** 1816-09

**Authors:** 


*For the London Medical and Physical Journal.*

### *On the Necessity of Accoucheurs;* by MOGOSTOKOS.

IN discussing the merits of the male and female practitioners of midwifery, it has been usual to argue, in favour of the sufficiency of the latter, that in most countries midwives only officiate upon such occasions, and yet that the parturient women, with few and rare exceptions, pass through the painful process speedily and safely. May it not, however, be doubted whether this argument is valid? Has the fact been proved that the process of child-birth is more safe and speedy than among us? I think not. We have, it is true, instances mentioned very abundantly of travellers through various countries, who have accidentally met with women speedily and easily delivered, and the same thing is happening in England every day; but is this all? are these travellers acquainted with the whole of the circumstances? I fear not. As in our country the frequent occurrence of easy labours does not prove that parturition is always safe, neither will the occasional instances met with by travellers lead a man of reflection to suppose that the doom, "in sorrow shalt thou bring forth children," has been suspended in other parts of the world, though still inflicted here.

Among the countries considered as more particularly favourable to the parturition of the natives, Italy, Spain, and Portugal have been frequently mentioned. Of Spain I have nothing to say; but the following extracts will, I think, go far to prove that in Italy and Portugal the most fatal consequences from child-birth are to be apprehended.

Signor Assalini, in his "Nuovi Stromenti di Ostetricia e loro Uso, 1811," gives a list of the number of women delivered in the hospital of St. Catherine, at Milan, during the year 1810.

The

The number admitted was 269—

| | |
|---|---|
| Natural and speedy labours ------ | 205 |
| Natural, but slow, ditto ---------- | 33 |
| Complicated labours ------------ | 27 |
| Impossible ------------------- | 4 |

so that more than one in nine cases was attended with irregularity and danger.

Of the women delivered ten died.

1 of apoplexy.*
1 of epilepsy.*
1 of hæmorrhage.
1 of metritis.
1 of rupture of the uterus.
2 of organic diseases.
3 in consequence of the Cæsarean operation.

Of the children, 228 were born alive, among which was one saved by the Cæsarean operation. 41 were dead.

This shews a dreadful mortality of women in child-bed, viz. 1 in 27! and a no less dreadful sacrifice of infant's lives, viz. 1 in 6½!

The other extract I have to bring forward is from a little work published in 1809, under the name of "A Picture of Lisbon, taken on the Spot, &c. &c. by a Gentleman many years resident at Lisbon"; and is as follows:

"Accoucheurs are hardly known in Portugal. They are proscribed by the manners and prejudices of the nation, and by the insinuations of the monks, more than by any real principle of modesty. Jealousy renders the husbands insensible both to the sufferings of their wives, for want of assistance, under the most trying circumstances of nature, and to the danger to which the life, as well of the mother as of the infant, is exposed, from the unskilfulness of the midwives. *The disastrous consequences that continually arise from the latter cause are such as loudly call for the interference of the legislature*, which, however, is hardly to be expected from a government like the Portuguese."

Upon the whole, I am inclined to believe that it will be as well for our females not to dismiss their present obstetric attendants till more complete proofs are produced of the competency of midwives to conduct the important process of child-birth; and be it remembered that the female are much more liable than the male practitioners to commit errors through hastiness and impatience.

*July* 29, 1816.

---

* Might not these be cases of puerperal convulsions?

